# Low-Field Electron Emission Capability of Thin Films on Flat Silicon Substrates: Experiments with Mo and General Model for Refractory Metals and Carbon

**DOI:** 10.3390/nano11123350

**Published:** 2021-12-10

**Authors:** Ivan Bizyaev, Pavel Gabdullin, Maxim Chumak, Vladislav Babyuk, Sergey Davydov, Vasilii Osipov, Alexey Kuznetsov, Olga Kvashenkina, Alexander Arkhipov

**Affiliations:** 1 Institute of Electronics and Telecommunications, Peter the Great St. Petersburg Polytechnic University, Polytechnicheskaya St., 29, 195251 St. Petersburg, Russia; ivanbiziaev@yandex.com (I.B.); pavel-gabdullin@yandex.ru (P.G.); equilibrium2027@yandex.ru (M.C.); vladislavbabyuk@gmail.com (V.B.); sergey.nik.davydov@mail.ru (S.D.); osipov1140@gmail.com (V.O.); kvol.spbspu@gmail.com (O.K.); 2 Chemistry Department, Institute of Physical Chemistry, University of Cologne, Greinstr. 4-6, 50939 Köln, Germany; 3 Nanotechnology Research and Education Centre RAS, Alferov St. Petersburg National Research Academic University, Khlopin St. 8/1, 194021 St. Petersburg, Russia; leshiy2698@mail.ru

**Keywords:** thin films, cold electron emission, molybdenum, carbon, refractory metals, electrically nanostructured heterogeneous materials and films, thermoelectric effects

## Abstract

Herein, we describe a study of the phenomenon of field-induced electron emission from thin films deposited on flat Si substrates. Films of Mo with an effective thickness of 6–10 nm showed room-temperature low-field emissivity; a 100 nA current was extracted at macroscopic field magnitudes as low as 1.4–3.7 V/μm. This result was achieved after formation treatment of the samples by combined action of elevated temperatures (100–600 °C) and the electric field. Morphology of the films was assessed by AFM, SEM, and STM/STS methods before and after the emission tests. The images showed that forming treatment and emission experiments resulted in the appearance of numerous defects at the initially continuous and smooth films; in some regions, the Mo layer was found to consist of separate nanosized islets. Film structure reconstruction (dewetting) was apparently induced by emission-related factors, such as local heating and/or ion irradiation. These results were compared with our previous data obtained in experiments with carbon islet films of similar average thickness deposited onto identical substrates. On this basis, we suggest a novel model of emission mechanism that might be common for thin films of carbon and refractory metals. The model combines elements of the well-known patch field, multiple barriers, and thermoelectric models of low-macroscopic-field electron emission from electrically nanostructured heterogeneous materials.

## 1. Introduction

In an increasing number of applications, thermal cathodes are being replaced by cold cathodes, which have the advantages of higher energy efficiency, faster response, and easier miniaturization. Field-effect cold cathodes are employed in electron microscopes, vacuum microwave devices [1,2,3], compact X-ray tubes [4,5,6], light sources [3,7,8], electron beam lithography systems [9], etc. In the vast majority of cases, such cathodes use metal needle-like tips [6,10,11], carbon fibers [3,7], or nanotubes [6,12,13] to enhance the electric field and, thus, to reduce the required voltage. However, the concentration of the electric field at the high-aspect-ratio surface features also implies the concentration of destructive factors such as ion sputtering, Joule heating, and ponderomotive forces. Consequently, long-term stability and lifetime remain among the key issues for cold cathodes with sharp emitting tips, which brings into attention planar or smooth-surface cold emitters as competitive options [2,6,11,14].

One of the possible ways to build a fully planar large-area cold emitter is represented by experimental MIM and MOS sandwich structures comprising an insulator/oxide film (I or O) of an nm-scale thickness deposited onto a metal (M) or semiconductor (S) substrate and covered with a thin metal layer [15]. Application of a voltage of the proper sign across the insulating layer leads to the injection of hot electrons into the top metal film. If its thickness is of the order of the electron scattering length or less, then a fraction of these high-energy electrons can travel to the vacuum boundary and be emitted. Results of early experiments did not seem very promising; the emission efficiency (ratio of emission current to full current through the system) was below 1% [16,17,18,19], the emission current was often unstable and nonuniformly distributed over the cathode area [17,18,19], it reached maximum values only after “forming” events resulting in the appearance of “defect channels” across the insulator film [18,19,20], and the best parameters were obtained with highly defective or even discontinuous metal top electrodes [20]. Thurstans and Oxley in their paper [21] identified the processes that occurred in those experiments as tunneling conduction through chains of metal islands produced in the insulator during the forming process and electron emission from the chain ends. Later, with refined technologies, the properties of MIM/MOS cathodes were significantly improved [22,23], while the emission mechanism apparently came into agreement with the original concept [15]. The most recent progress was related to the idea of using graphene (G) films as top electrodes [24,25,26]; Murakami et al. reported on the achievement of emission efficiency up to ~50% and current densities >100 mA/cm^2^ with the GOS structures [26].

Another type of smooth-surface cold emitters employs discontinuous films consisting of separate metal islands on insulating substrates [27,28,29,30]. Electron emission occurs under the action of a lateral current flowing along the film between two metal contacts deposited on top of the substrate, but only after the electroforming procedure produces “current channels,” i.e., percolation paths in the film. Emitted electrons originate from discrete emission centers (EC), one per current channel. The model of this phenomenon suggested by Fedorovich, Tomchuk et al. [29,30,31] has a definite similarity with the mentioned model by Thurstans and Oxley [21], with adjustments for a different current direction; tunneling between metal islands produces hot electrons capable of emission into a vacuum. Authors of the model [29,30,31,32] state that emission may be enhanced by confinement effects in nanosized metal islets, leading to decoupling between electrons and lattice vibrations. As a result, the energy loss rate for hot electrons in the nanoparticles can be approximately 100 times lower than in bulk material, with a corresponding increase in the electron temperature—whereas the lattice temperature of the EC islet remains relatively low [30,33], which ensures its integrity. Validity of the proposed model was confirmed by (1) optical emission accompanying the emission of electrons and originating from the same centers [27,30] and (2) emission (both electron and optical) from the same films energized with visible [34], infrared [29,30,34], or microwave [35] radiation, which could also produce long-living hot electrons in the nanoparticles.

Along with metal ones, carbon island films are also capable of electron emission under the action of the lateral current [29,36]. Moreover, it has been suggested (e.g., in [37]) that even in the case of metal films, the ECs might represent small carbon islets produced from hydrocarbon contaminants by cracking in the electric field during the forming process. Carbon islets are most resistant to high temperatures, and therefore, they may be the most effective as the ECs.

In our previous works [38,39,40], we investigated low-macroscopic-field (LMF) electron emission from island carbon films on silicone substrates coated with a native oxide. Those smooth-surface structures had much in common with the structures discussed above, but in our experiments, no energizing current was passed either through or along the emitting structure. Nonetheless, cold electron emission was induced by an electric field with a macroscopic magnitude as low as 1 V/μm or less, i.e., of the same order as the field employed for extraction of emitted electrons in the experiments where the highest emission efficiency values were obtained with planar MOS or GOS sandwich structures (5 kV/10 mm in [41], 1 kV/5 mm in [26]). Emissivity of the tested films emerged or improved after an electrothermal forming treatment. The LMF emission capability is known to be inherent in various electrically nanostructured heterogeneous (ENH, [42]) carbon species, such as diamond or diamond-like films, nanographites, and *sp*^2^/*sp*^3^ composites [2,42,43,44,45,46,47,48,49,50,51,52], even in the absence of visible sharp protrusions of the outer boundary. Very different models [14,42,43,44,45,51,52,53,54,55,56,57,58] were proposed to explain this phenomenon. Many of them were based on the assumption of the field penetration into the emitter or/and its enhancement at internal conductive structures, such as particles of *sp*^2^-bonded carbon, grain boundaries, or electroformed current channels. However, neither of these models could be relevant to the case of LMF emission from nm-thick carbon islets on Si with native oxide having a thickness of only several nanometers because all layers of the system are too thin and the penetration of an external electric field with a magnitude of the order of 1 V/μm into this structure cannot produce sufficient potential differences, comparable (in compatible units) with the work function. To explain the observed LMF emission from such films, we proposed a model that combined elements of the known models of lateral potential variation (“patch field”) [45,52,59,60] and hot-electron (or dual-barrier) emission [49,61], and it also accounts for specific nanoscale thermoelectric effects in the article [62]. Our further experiments were aimed at the verification of this model via testing of the LMF emissivity of metal films grown on identical substrates as the carbon films in [38,39,40]. Early results of these experiments were reported in [63]. Among studied metals (Mo, W, Zr, Ni, and Ti), films of molybdenum showed the best LMF emissivity.

This paper presents a more comprehensive experimental investigation of LMF emissivity of molybdenum films on flat silicon substrates. Results of these experiments forced us to revise the emission model proposed in [62]. We hope that the revised model may be relevant for both metal and carbon films.

## 2. Materials and Methods

### 2.1. Sample Preparation

Samples of metal thin films were grown by magnetron sputtering on flat substrates of crystalline silicon with native or, on several occasions, specially grown oxide layers. In most instances, the substrate plates, approximately 1 × 1 cm^2^ in size, were cut from boron-doped wafers with 10 Ω⋅cm resistivity, the type “KDB-10” in the Russian denotation. They were preliminarily purified with isopropyl alcohol and rinsed in distilled water. Films of Mo were grown in a Mantis HEX deposition system (Mantis Deposition, Thame, UK) equipped with a 2” dc magnetron sputter source. Targets of 99.99% pure metal were used; the system had a base pressure of 10^−6^ mbar. Up to six substrates were installed on a rotating stage at the distance of 180 mm from the target and baked at 150 °C in high vacuum for 10 min or more for degassing. To remove target surface contaminants before starting the actual deposition, each target was sputtered for 5 min with substrates shielded from the sputtered matter with a shutter. Sputtering was carried out in an argon atmosphere at the pressure 1–5 × 10^−3^ mbar with the substrate temperature chosen in the range 100–150 °C. Film growth rate was controlled in the range 0.1–1.0 Å/s via sputtering power (50–150 W). Effective (average) thickness of deposited films was determined by a quartz balance that was calibrated by measuring the thickness of a Mo film with the balance and using a LYRA SEM (Tescan, Brno, Czech Republic) with an ion beam column.

### 2.2. Field Emission Testing

Emission properties of film samples were investigated with the use of a custom-built setup mounted in a TSN-2 vacuum chamber (SPbPU, St Petersburg, Russia) with two sputter ion pumps maintaining a residual pressure of 1 × 10^−9^ mbar. Up to six tested specimens (film samples on substrate plates) were mounted into identical testing cells, each equipped with a separate heater allowing temperature variation in the range 20–600 °C. First, the specimens were degassed at 150 °C. Emission properties were probed in diode arrangements with parallel-plate field geometry; the field was produced in gaps of *d* = 0.6 mm width between each specimen and an opposite molybdenum cylindrical flat-top anode. The spacers used to set the gaps’ width were removed before evacuation of the setup (which had sufficient mechanical rigidness), so the insulation in the cathode-anode gap was purely vacuum. Diameter of the anodes (6 mm) was chosen to be less than the specimens’ lateral size to exclude the current from the sharp edges of the substrate plates. Emission current-voltage (I–V) characteristics were measured by applying a positive potential to the anode with respect to the grounded specimen. Potential *U* was linearly increased from zero to a chosen maximum value (not higher than 4.5 kV) in 35 s and then ramped down at the same rate. This gave us two branches of the I–V characteristics; the measurement was repeated several times to check reproducibility of the result.

### 2.3. Surface Characterization

The film samples were characterized before and/or after emission tests to assess the effect of current extraction on their morphology. For this purpose, we employed scanning electron microscopes (SEM), including models LYRA, MIRA, and SOLARIS (Tescan, Brno, Czech Republic). Energy dispersive X-ray spectroscopy (EDS) units of these instruments provided information on the elemental composition of studied spots. The surface topography with 3D-resolution was taken using an atomic force microscope (AFM) Nano-DST (Pacific Nanotechnology, Santa Clara, CA, USA) operated in semi-contact mode under ambient conditions. The images were processed with the Gwyddion software.

Fine morphological details were examined using a vacuum scanning tunneling microscope (STM) SM-2000-Vac (Proton-MIET, Moscow, Russia) in the constant current and constant height modes. STM probes were made by mechanical cutting from polycrystalline Pt/Ir wire. To obtain finer STM images, the probes were additionally sharpened by ion etching in a two-beam Tescan SEM and by in situ high-current treatment in the STM. Surface topography was imaged in the constant current mode; local density of states (LDOS) was mapped in the mode with modulation of the bias voltage. The SM-2000-Vac instrument was also employed to study electronic properties of the surface by the method of scanning tunneling spectroscopy (STS); LDOS distributions were calculated from the measured tunneling I–V characteristics. On several occasions, an UHV VT AFM XA tool (Omicron Nanotechnology GmbH, Taunusstein, Germany), integrated in a NanoLab platform, has been used for the same purpose.

Raman spectra were collected from several samples using a Horiba Jobin-Yvon LabRam HR800 spectrometer with 532 nm laser excitation at a low power level to exclude heating effect.

## 3. Results

### 3.1. Emission Properties

A major part of the tested thin Mo film samples manifested an LMF emission capability, more or less pronounced. Coatings with an effective thickness (i.e., the one determined by the quartz balance method) of 2 nm and 4 nm showed emission with low thresholds (U = 1–2 kV), but their emissivity was unstable and decayed in several hours or days. No emission current in the available voltage range U < 4.5 kV was obtained from a sample with a much thicker 20 nm Mo layer. The best emission properties were shown by films with a thickness between 6 and 10 nm.

**Figure 1:**
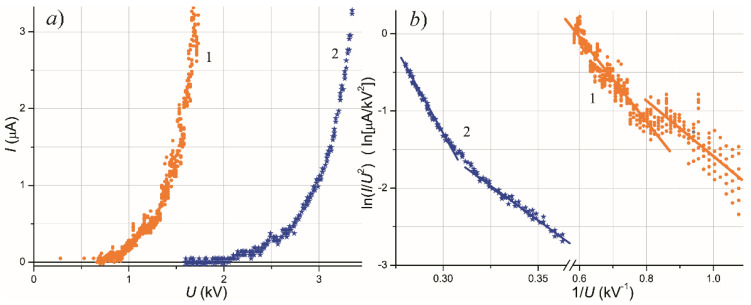
(**a**) I-V characteristics of the room-temperature emission for Mo film samples with an effective thickness of 10 nm (1) and 6 nm (2). (**b**) The same dependencies in the FN coordinates. Cathode-anode distance d = 0.6 mm, anode diameter 6 mm, and planar field gap geometry. The characteristics were measured after activating treatment of the samples.

Figure 1a presents I–V emission characteristics (current I with voltage U) for the two samples, with 10 nm and 6 nm Mo coating, which were chosen for detailed further study. Values of the threshold macroscopic field U/d (at 100 nA) for these characteristics are remarkably low, 1.4 and 3.7 V/μm, respectively. Being plotted in the Fowler–Nordheim (FN) coordinates ln(I/U^2^) vs. 1/U (Figure 1b), they (or at least their segments) can be approximated with reasonable accuracy by straight lines, which is often considered as a confirmation of the tunneling nature of the emission mechanism. However, the slopes of the FN characteristics in the assumption of a flat emitting boundary (field enhancement factor β = 1) correspond to extremely low work function values φ_*FN*_ = 10–50 meV, and even taking the highest plausible estimate of field enhancement β = 10 for the surface topography seen in microscopic images (in Section 3.2), we obtained φ_*FN*_ values no greater than 0.25 eV.

(It is recognized now that the FN formula for field electron emission (FEE) is not fully correct [64], and more precise equations were proposed [65,66,67]. However, for comparison with the data obtained in this study, the accuracy of the FN theory seems sufficient, and the conceptual apparatus associated with it is still more familiar to many of the researchers [68], which may justify its use herein.)

It is important to note that only a minor part of the tested films possessed the LMF emission capability in their pristine, as-grown state. For other samples, this property was developed via a special preliminary treatment by the combined action of temperature and electric field, similar to that previously employed for the activation of nanocarbon film emitters [69]. The thermal-field (TF) treatment procedure optimized for Mo films consisted of the following steps.

First, electric field of a macroscopic magnitude ≤ 1 V/μm was applied to the sample at room temperature. Then, the temperature was ramped up at the rate of ~5 °C/min until an emission current appeared and reached the value I_TFT_ = 100 nA, or to 600 °C (at higher temperatures, the samples usually produced a noticeable thermionic current concealing the field-induced component). The sample was kept at the attained temperature for 30 min while the current was stabilized at I ≈ I_TFT_, which usually required a gradual reduction of the applied voltage. Thereafter, the temperature was slowly (~2 °C/min) decreased to the ambient one. At this step, the extracted current was also maintained at ≈I_TFT_ by voltage adjustment.

**Figure 2:**
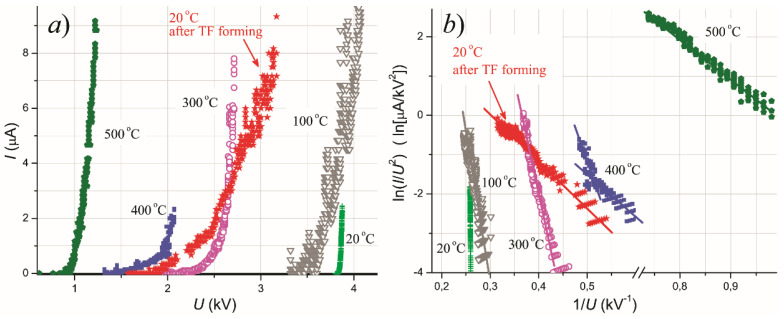
(**a**) Transformation of I–V emission characteristics of a 6 nm Mo film sample in the course of its forming. Curve “20 °C” reflects emission properties of the sample before the procedure. Then, the temperature was successively raised to 100, 300, 400, and 500 °C, and the corresponding plots are shown. Curve “20 °C after TF forming” illustrates the treatment result. (**b**) The same characteristics re-plotted in the FN coordinates.

The characteristics depicted in Figure 1 were measured after the TF conditioning of the samples. Figure 2 presents a set of emission I–V plots acquired in the course of TF procedure applied to the 6 nm-thick Mo film. The treatment resulted in the reduction of the turn-on voltage from 3.8 to 1.8 kV; compare the curve labeled “20 °C” reflecting the initial state of the emitter and the curve “20 °C after TF forming” for its activated state. Effective work function (calculated in the assumption of β = 1 from the slope of the characteristics in FN coordinates shown in Figure 2b) also decreased from 0.4–0.5 eV to 20–25 meV.

In general, the described TF activation turned out to be most effective for the Mo films, as it caused a decrease in the threshold field several times and an increase in the emission current by at least an order of magnitude. A number of simplified routines were also tested—for example, a purely thermal treatment without the pulling field or with the field turned off during the specimen cooling. However, these attempts were unsuccessful and resulted in emissivity extinction. Contribution from the “field component” in the forming efficiency may be associated with a flow of ions irradiating of the emitting regions that can facilitate local film reconstruction.

**Figure 3:**
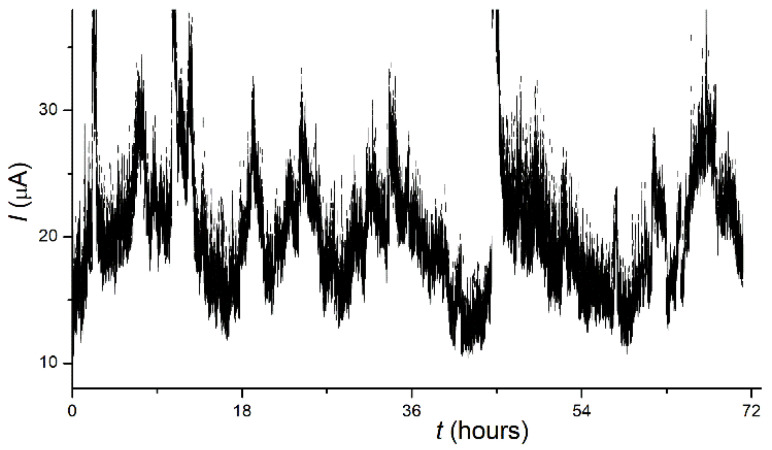
Time dependence of current (I–t plot) at a constant U for a 10 nm Mo film sample.

Figure 3 depicts a plot of emission current vs. the time for a TF-activated 10 nm Mo film. The graph shows that the sample produced an emission current with a mean value near 20 μA for the period of 72 h with no signs of degradation. However, the current fluctuations were as large as 30–50% of the mean value, and their magnitude also remained approximately constant over time. A similar situation was considered in the book [2] (p. 268) as an indication that the current oscillations “were intrinsic to the emission mechanism”.

For other samples and at higher values of the extracted current (>100 μA), deterioration of the emissivity was observed with characteristic times of the order of hours. Together with the observed dramatic effect of TF treatment, this supports the conclusion that the emitting structures of the studied films were strongly influenced by emission-related factors such as local heating and/or ionic irradiation.

### 3.2. Surface Morphology

#### 3.2.1. Pristine Films and Areas

Microscopic studies, carried out by several different methods, have shown that the films that were not exposed to an electric field had a fairly smooth and uniform surface. This applies equally to pristine samples (i.e., imaged before the emission tests) and to specimens’ peripheral areas outside of the anode “footprint,” which remained unaffected by the field during the testing.

**Figure 4:**
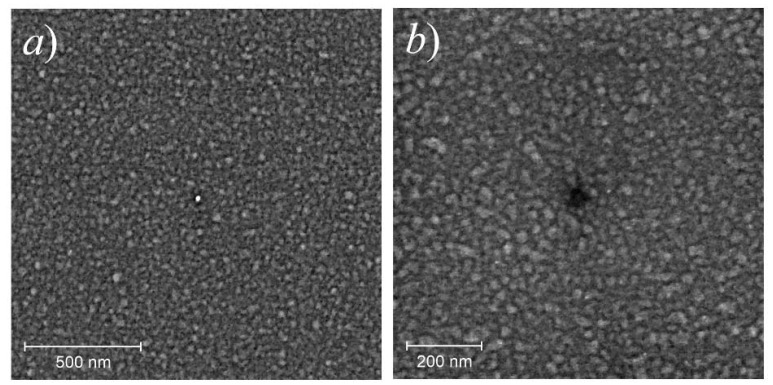
SEM images (in-beam SE detector) of pristine Mo samples with an effective thickness of 10 nm (**a**) and 6 nm (**b**). The central features were produced by the probing electron beam.

Before emission testing, the coatings were continuous. The height ranges in AFM topography profiles were several times less than the thicknesses determined by the quartz balance method. SEM images shown in Figure 4 demonstrate that the films comprise grains with transverse dimensions, 10–50 nm, presumably representing crystallites connected through an amorphous matrix. EDS elemental maps showed a practically uniform distribution of the deposited metal, oxygen, and silicon over the surface. The presence of the latter can be explained by the small thickness of the film, below the penetration depth for the probing electrons. Oxygen, which has been detected in significant quantities, can either be incorporated in the native silicon oxide layer preserved on the substrate or bound to the metal during sample transfer through the atmosphere, or both; the resolution provided by the EDS unit was insufficient for the reliable localization of oxygen in the pristine films.

**Figure 5:**
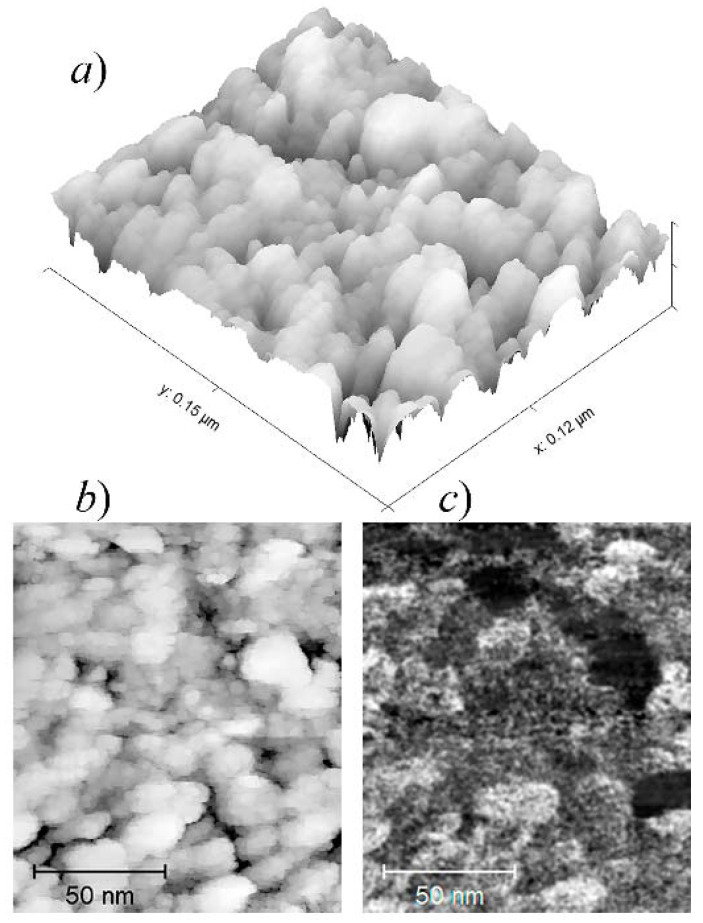
(**a**,**b**) Surface topography images for an as-fabricated 10 nm Mo film obtained by an STM operated in the constant current mode; (**c**) a local DOS map for the same area. The dark spots presumably show electrically separated Mo grains.

Parts (a) and (b) in Figure 5 display STM surface topography images for a typical pristine Mo film. Nanosized grains similar to those depicted in the SEM images in Figure 4 can be seen here in higher resolution. In the LDOS map for the same area presented in Figure 5c, some of these grains are shown as dark spots. This peculiarity may reflect the difference in their electronic properties (i.e., conductivity) or more likely, poor electrical contact of these special grains with the substrate and with the rest of the film.

#### 3.2.2. Raman Studies

**Figure 6:**
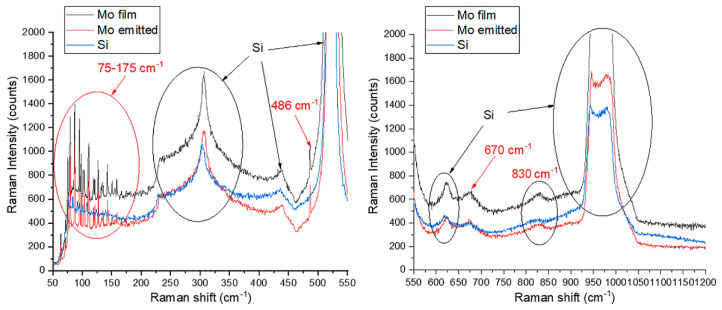
Raman spectra in the bands 50–550 cm^−1^ and 550–1200 cm^−1^ for 10 nm Mo film samples before and after TF conditioning and emission testing, in comparison with a spectrum for a clean substrate of the same type (p-Si KDB-10).

Figure 6 presents a Raman spectrum recorded with the 10 nm Mo film after its TF conditioning and emission testing—the sample showed LMF emissivity characterized by the threshold field value of approximately 3 V/μm. This spectrum is compared with the spectra measured for a clean substrate and for a similar film in the as-grown state. The most prominent features in all these spectra are common and relate to silicon substrate [70], which is natural for optically transparent films. The group of peaks at 75–175 cm^−1^ is noticeable in the spectra of both coated specimens and is absent in the spectrum of the substrate. Such a group is known to be a characteristic of various modifications of molybdenum oxides, including crystallohydrates [71,72]. Other features that distinguish spectra of Mo films from those of a clean substrate are a narrow peak at 486 cm^−1^ and, less confidently, broader maxima near 670 and 830 cm^−1^. According to the literature [71,72], these peaks can be attributed to the thermodynamically stable orthorhombic phase of molybdenum trioxide [73]. Thus, the assumption of partial oxidation of the metal finds experimental confirmation. The oxide shells, formed around the metal particles, can impair the mechanical and electrical coupling between the grains, as it was probably revealed by the STM images in Figure 5.

The spectra in Figure 6 show no definite difference between pristine and activated films, which suggests that the TF activation of the emission is not achieved through changes in chemical bonding.

#### 3.2.3. Effect of Forming

**Figure 7:**
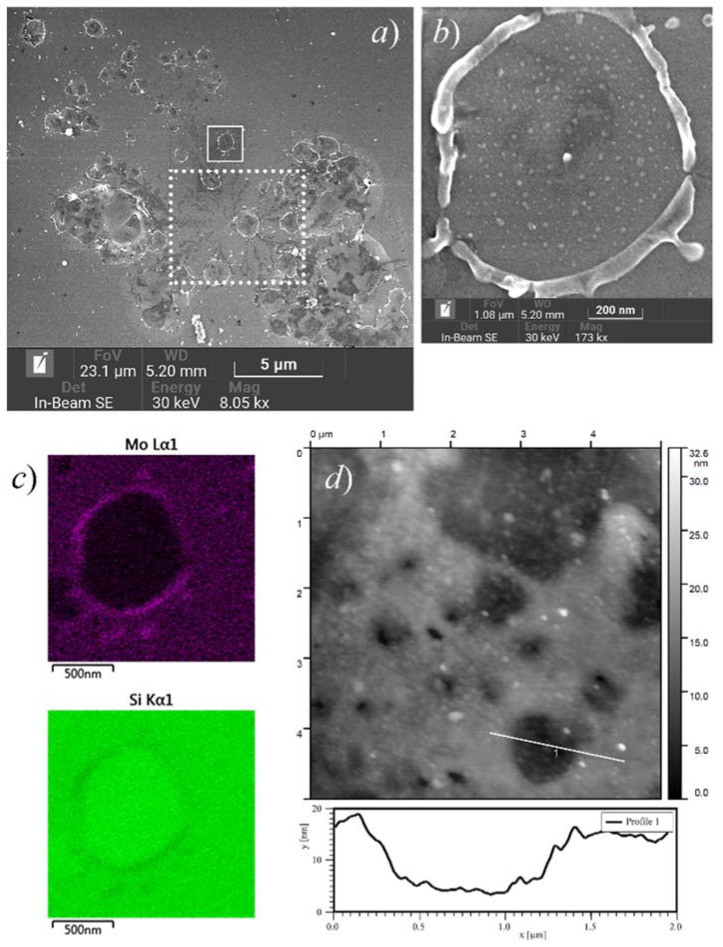
Large-scale features found on the surface of a 10 nm Mo film sample (the same as in Figure 4a) after emission experiments: (**a**) an overview SEM image; (**b**) magnified SEM image of a selected μm-scale hole in the Mo coating; (**c**) EDS elemental maps (Mo and Si) for the same area; (**d**) similar holes in an AFM image, with topography profile across one of the holes in the bottom graph.

The extraction of the emission current and TF forming had a notable effect on the morphology of the films. Damaged (or reconstructed) areas were visually detected on the emissive samples after their testing. Figure 7a shows an overview SEM image of such an area on the 10 nm Mo film sample recorded after the 72-h durability test described above (Figure 3). Analysis of this and other microscopic images revealed several types of newly formed morphological features.

Some of them can be attributed to arcing events that occurred during emission experiments—such field gap breakdowns, interrupted by the action of a protective resistor in the anode circuit, can be recognized in the I–t plot in Figure 3. Among the features seen in Figure 7a, the arcing may be responsible for the dendritic star-shaped film rupture enclosed in the dotted box. The absence of similar features in the immediate vicinity suggests that the arcing itself did not create the conditions for repeated breakdowns, i.e., it did not produce features capable of LMF electron emission.

Features of another type are the circular crater-like holes in a metal coating with a transverse size of the order of 1 μm. In Figure 7a, one of them is marked with a solid-line box; its SEM image in higher magnification is given in Figure 7b. The EDS elemental maps in Figure 7c show that the raised rim and the droplets surrounding the hole are made of Mo (and not of Si) and incorporate metal from the bottom of the hole, where only a small quantity of the film material remains in the form of nanoscale islands. Figure 7d presents an AFM image of a similar sample area and a topography profile across one of the holes. Its depth (≈10 nm) corresponds to the film’s initial thickness, and the bottom is approximately flat. Thus, the hole only pertains to the film and does not affect the substrate. This combination of properties suggests that the holes do not represent craters left after electric explosions, but they were formed as the result of less catastrophic processes that probably involved lateral transfer of the film material by capillary forces at relatively large (μm-scale) distances.

**Figure 8:**
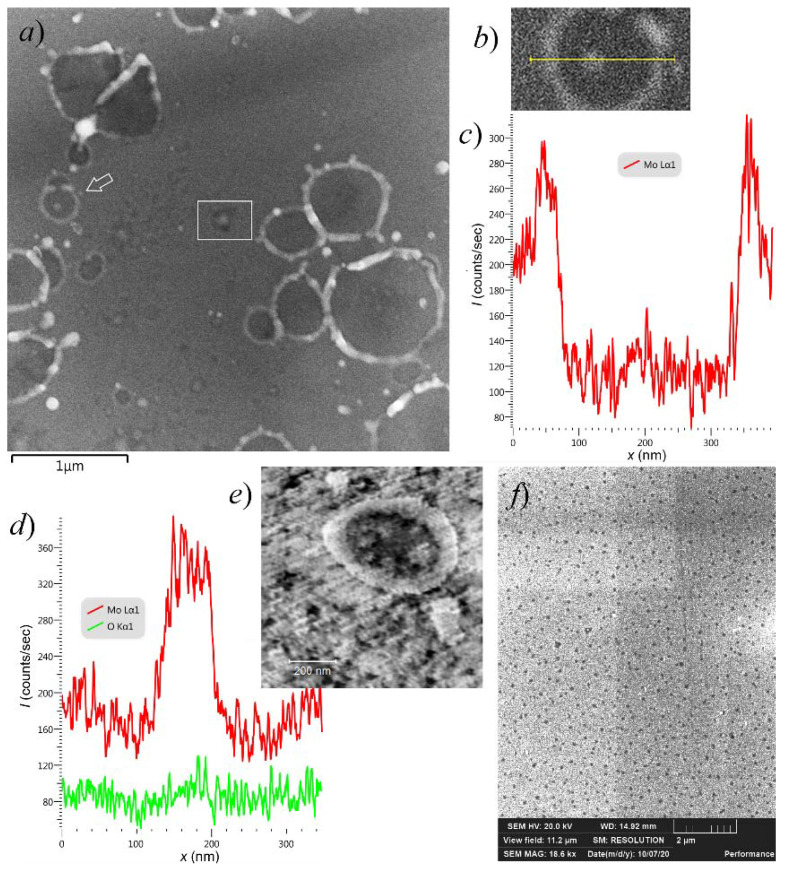
Sub-micrometer breaches in the emissive 10 nm Mo film (the same as in Figure 7): (**a**) an overview SEM image showing the sub-μm holes along with the larger ones; (**b**) magnified SEM image of a typical sub-μm hole with elevated rim and central hillock (shown with arrow in part (**a**)) in the Mo coating, (**c**) EDS elemental profile (Mo) across this hole (missing the central hillock) shows the presence of an excessive amount of metal in the rim; (**d**) elemental profiles (Mo and O) across a hole and its central hillock, and the hole is indicated with the box in part (**a**); (**e**) STM image of a similar sub-μm hole, the scale bar is 200 nm; (**f**) sub-μm defects in a SEM image of an area away from large craters, and the rectangular darker and lighter areas are the result of repetitive SEM imaging.

The assumption of gradual growth of the film defects can be supported by the observation of such features having different sizes, presumably passing through different stages in their growth. In the SEM images in Figure 8a, the micron-scale features are surrounded by smaller holes with typical dimensions of the order of 100 nm, many of them with central hillocks. As the larger holes, the smaller ones have elevated rims (Figure 8b,c). The EDS data displayed in Figure 8d demonstrate that oxygen is distributed across such a hole almost uniformly, while Mo concentration (parts c and d of the Figure show its profiles away from the central hillock and through the central hillock, respectively) corresponds to the hole topography. Therefore, the EDS-detected oxygen is not the one bound in the molybdenum oxides revealed by the Raman spectra, but rather the oxygen incorporated in the silicon dioxide layer, which remained unaffected by the processes resulting in the hole formation. This leads to the conclusion that the degree of oxidation of the film cannot be high and that Mo is present there predominantly in metallic form.

Figure 8e displays an STM image of a similar sub-micrometer rimmed hole with islets of the remaining film material within. Apart from the relatively large (>300 nm) rimmed feature, the image also shows smaller (<100 nm) film ruptures having irregular shapes. On activated Mo film samples, such pores were found over the entire area exposed to the electric field (Figure 8f), but not on film margins outside the anode “footprint”. They were not seen on pristine films as well. Therefore, we relate the appearance of such defects to the combined action of the electric field and temperature during the TF conditioning. Some of them could possibly have served as the nuclei that further developed into the sought LMF emission centers.

**Figure 9:**
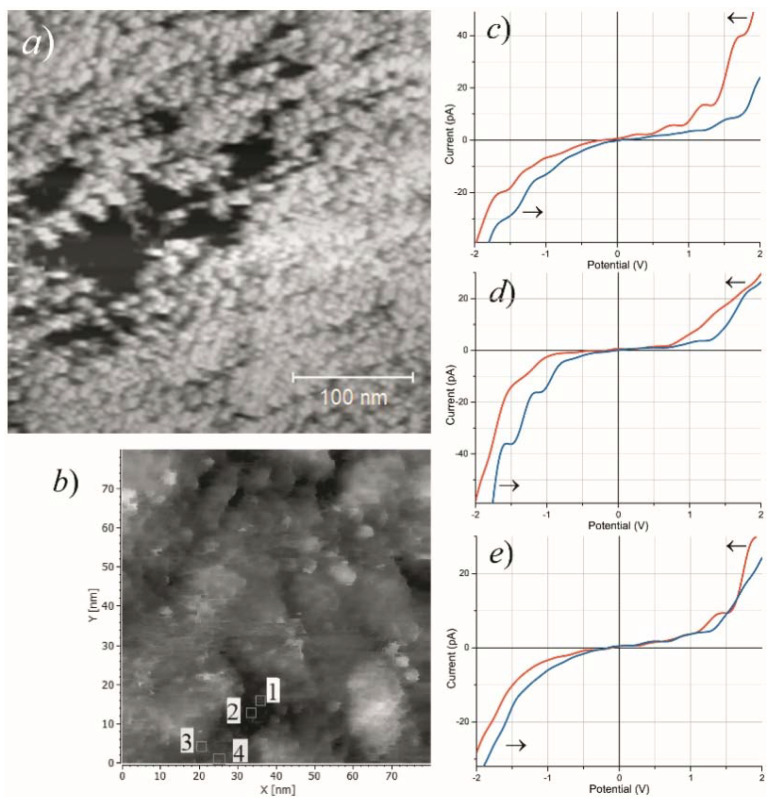
(**a**,**b**) STM images of film ruptures in a conditioned 10 nm Mo film; (**c**–**e**): STS spectra measured at positions 1, 2, and 3 marked in image (**b**), and arrows indicate voltage sweep directions.

STM images of the small angular pores in the highly emissive Mo film are shown in Figure 9a,b. The plots in this figure present STS current characteristics measured at different points inside and near one of the pores, which reveal dissimilar local electronic properties. The graph in Figure 9d displays a bandgap in DOS distribution of approximately 1 eV width near Fermi level—apparently, the probe was in contact with semiconductor substrate at the hole bottom. The plots in Figure 9c,e show finite DOS at the zero point, inherent in conductors. All these spectra include staircase-like features, which are usually associated with nanoparticles that have isolated electron systems. In literature, they are attributed either to dimensional quantization [14,57,74] or the Coulomb blockade effect [2,30,75,76,77,78,79]. Thus, it can be inferred that at least some islets directly observed in microscopic images (Figure 7b, Figure 8e and Figure 9a) were insulated from their environment—as it is required by several emission models [2,42,43,44,49,50,51,52,53,54,55,56,57], including the model proposed in [62].

## 4. Discussion

### 4.1. Comparison with Literature Data

In addition to establishing the very fact of LMF emission from thin films of a refractory metal, the acquired experimental data can be useful for better understanding of the LMF emission mechanism. For this purpose, they should be compared with literature data on cold electron emission from thin metal films and with our own data of LMF emission of electrons by thin carbon films deposited on identical substrates and tested by the same methods as the metal films in the present work.

An important difference between our previous experiments with thin carbon films [38,39,40] and the present work consists in different structures of as-fabricated films. The carbon films studied in [38,39,40] initially comprised nanoislands, whereas in this work (and in [63]), due to technological limitations, we deposited continuous films of metals. The process of agglomeration of flat thin films deposited on non-wettable substrates is known as solid-state dewetting [80,81]. The shapes of the holes in Mo coatings (Figure 7 and Figure 8) were very typical for the result of dewetting based on surface diffusion and capillary energies; they had internal hillocks, elevated rims, and surrounding “web-like or branched structure” [81,82] that accumulated a part of the film material removed from the holes. Disintegration of films into particles is known to occur at temperatures well below melting points of film materials [80]. In [82], the observation of dewetting of molybdenum films at hour-scale timeframes required them heating to the temperature of 940 °C, substantially higher than the range of TF treatment in the present work. However, the agglomeration process can be stimulated not only by temperature but also by electron, optical or ion irradiation [83,84,85], i.e., the factors that can be associated with emission. The effect of a prolonged action of electron beam can be illustrated by the SEM images in Figure 4, where the central feature most probably resulted from the electron-induced film reconstruction. Electric field can also increase atomic mobility and thus may control the dewetting process [81].

At this point, we can find an obvious analogy between our results and the literature data on current-induced emission from islet films [27,28,29,30] and from MIM/MOS sandwich films [18,19,20]. In those works, effective and stable emission also appeared only after electroforming procedures, and the emission sites were identified with film features or defects produced by the electroforming. For instance, the SEM image of a circular hole in the conditioned Mo film shown in Figure 7b has a notable similarity with a hole in the top metal layer of the planar MOS structure depicted in Figure 8 in the old paper [20], which may suggest a similar mechanism of formation of these features. Both Thurstans and Oxley [21] and Fedorovich, Tomchuk et al. [29,30,31] (for different types of cold emitters) explained the enhancing effect of electroforming by the emergence of separated nanoparticles. Quantum confinement effects can inhibit energy exchange between electrons and lattice vibrations, thus promoting the growth of hot electron lifetime and population. In our work, the presence of nanoparticles in the samples after their forming was confirmed by different experimental methods. Thus, the observed emissivity activation by the action of heating and electric field implies a connection between the LMF emissivity and formation of separated metal islets. Moreover, we can assume that the LMF emission centers are located at the junctions between regions with agglomerated and continuous film structure. The SEM overview image in Figure 7a demonstrates that many of the circular rimmed holes in the emissive coatings are organized in groups, and their shapes, distorted by overlaying, reveal the sequence of their formation. A careful examination showed that the centers of many newly formed craters were tied to the rims of the holes that existed at the time of their appearance. It seems natural to associate such points with the ECs that were active for some period of time.

Comparison of the results of this work with the data of many literature sources describing the cold emission of electrons from metal thin films is complicated by an important difference in experimental conditions. In our experiments, no additional stimulation of hot electron production (such as surface current or IR radiation [27,28,29,30,31]) was employed. However, LMF emissivity of nanogranular metal thin films manifested without any additional energizing had been previously reported, e.g., by Purohit et al. [86,87]. Moreover, several of the early experiments with MOS sandwich films discussed above employed relatively high magnitudes of extracting electric field applied to emitting structure surface: 5 kV/10 mm in [41], 1000 V/5 mm in [26], etc. This is comparable with threshold field values for the best samples tested in the present work, and the emission, observed in those experiments, could have been caused, at least partially, by the direct action of the applied field, not by the feeding current. LMF emission capability has also been reported for composite metal/carbon films, e.g., in our previous paper [88]. However, the LMF emissivity phenomenon is best studied for purely carbon ENH materials and films. Therefore, it might be useful to check whether the models proposed for ENH carbons agree with the experimental data of this work.

In the simplest approach, FEE is considered as a single step tunneling of electrons through a surface barrier. Quantitative description is given by the classical FN law [2,89] (for a flat 1D barrier) or by several revised relations [65,66]. However, the values of threshold of electric field and I–V dependency slopes in the FN coordinates, predicted by these theories, are much greater than those determined in our experiments. In principle, such discrepancy (not unusual for many nanocarbon forms) could be explained by a geometric field enhancement at protrusions of the emitter outer boundary. Assuming the tabular work function values for film and substrate materials (4.2 eV for Mo, 4.8 eV for Si), the field enhancement factor can be estimated from the slopes of the I–V plots in Figure 1b as β = 400–8000. Such β values are typical, for instance, for carbon nanotubes characterized by very high geometric aspect ratio. On several occasions described in literature, such morphological elements were found as an impurity in effectively emitting nanographite films [90,91]. However, in the reported experiments with metal films, no whiskers or other high-β features were found in SEM, AFM, or STM images recorded either before or after conditioning and emission testing.

Another possible explanation of LMF emissivity within the classical FEE paradigm might employ the suggestion of lowered work function of the emitter or some areas at its surface. Although, the slopes of the I–V plots in Figure 1b with realistically assumed β values (no more than 10) imply work function as low as 50–350 meV. The presence of such areas would result in thermionic emission at low temperatures; the Richardson–Dushman formula gives thermionic current density of the order of 10^7^ A/m^2^ (i.e., 100 nA/nm^2^) at 500 °C for 100 meV work function, which was not observed during the TF treatment of the samples.

Alternative emission mechanisms (different from direct electron tunneling via a single surface barrier) suggested for ENH carbon species [14,42,43,44,45,51,52,53,54,55,56,57,58,59] often associate the LMF emissivity with nanoscale heterogeneity. Internal boundaries and high gradients of physical parameters produce favorable conditions for emission facilitation via local field enhancement, generation of hot carriers, resonance, quantum size effects, etc. However, as it has been noted in the Introduction, some of these mechanisms cannot be realized in films and structures of nm-scale thickness. In [39,62], we have proposed a special emission model for islet carbon films deposited on oxidized silicon wafers. The new data on LMF emission from metal thin films force us to revise this model.

**Figure 10:**
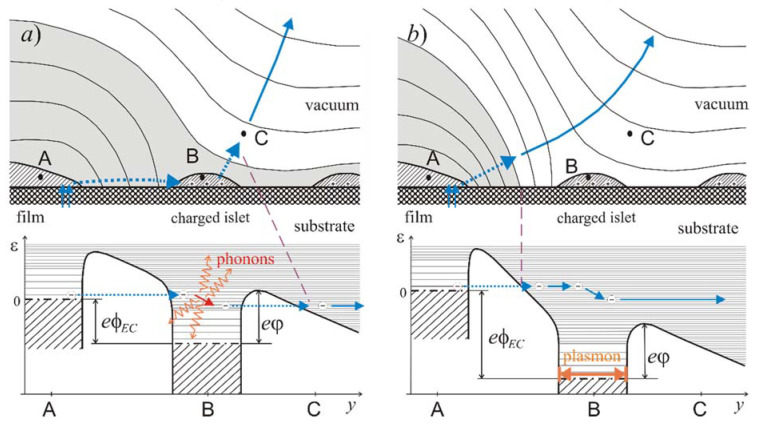
To the suggested LMF emission models: (**a**) the model in its original form; (**b**) the revised version, in which gray color marks the position of the potential barrier for electrons coming from the emitter’s Fermi level. In the bottom: energy diagrams along electron transfer paths; φ_*EC*_ is the islet potential, and *e*ϕ is film material work function.

According to the model [62] (Figure 10a), LMF electron emission is facilitated by lateral non-uniformity of surface potential, as large as several Volts across nanometer-scale gaps between adjacent film fragments. This non-uniformity originates from the thermoelectric effect powered by a heat released in electrically separated film islets serving as ECs. A semi-quantitative consideration, performed in [62] for typical parameters of the carbon islets, provided an estimate of the thermal flux density at the EC/substrate boundary as high as 1 MW/cm^2^. This flux is carried from the hot EC islet into the cold substrate by phonons [92]; their mean free path in crystalline Si at room temperature is 200–300 nm [93,94], i.e., much greater than the characteristic of the dimension of the considered system—islet lateral size ≈10 nm. Consequently, the flux has a ballistic character. This quality can dramatically boost the phonon drag contribution into the overall thermoelectric coefficient [62,95,96], thus providing the Volt-scale islet potentials (φ_*EC*_ in Figure 10) required in the described emission mechanism. The energy feeding the process eventually comes from the source maintaining the cathode-anode potential difference. In the model [62] illustrated by Figure 10a, a joint action of the external field and thermoelectric potential caused a tunnel injection of electrons into the positive EC islet with high (eV-scale) energies above the local Fermi level, determined by the potential drop across the tunnel junction. For such electrons, the probability of passing the surface barrier is high, and they can be effectively emitted into a vacuum. Emission efficiency could be hampered by electron-phonon and electron-electron scattering, resulting in rapid hot electron relaxation. However, the electron-phonon coupling in nanoparticles can be drastically reduced by quantization effects, as it has been theoretically justified in the mentioned articles [29,30,31,32] and in many other sources [97,98,99] (even while some publications [100,101] reported on the opposite tendency as well). In the special case of graphitic nanoparticles, the electron-phonon coupling is known to be inherently weak [102,103]. The electron-electron interaction of high-energy hot electrons may also be relatively inefficient due to specific features of this material, namely, the low intrinsic charge carrier concentration and large effective mass mismatch between different zones. The density of states in carbon near the normal position of the Fermi level is low, but it has strong maxima closer to the vacuum level [56,104,105], including the peak at the position of the σ* zone bottom. Hot electrons, injected into a carbon nanoparticle with energies above a DOS peak, may rapidly lose a part of this energy, which would be eventually converted into heat. However, the relaxation process must slow down as soon as an electron reaches a low-DOS region—due to the so-called “phonon bottleneck effect” [97,98,99]. From such high “metastable” levels, electrons may be efficiently emitted. As the result, LMF emission in the model [62] is accompanied by the generation of substantial heat power in the EC (of the order of 0.1 eV per emitted electron), which is necessary for the maintenance of sufficiently high thermoelectric potentials.

Assuming that the above LMF emission model [62] (Figure 10a) is fully relevant, one would expect a significant difference in emissivity between carbon and metals. Electron concentration in metals is much higher than that in graphitic carbon, which makes the electron-electron scattering an effective channel of energy re-distribution within the electron subsystem of a nanoparticle [106]. Consequently, non-equilibrium electrons injected into a metal particle must rapidly lose major part of their excessive energy to thermalize, even if the electron-phonon interaction is hindered (in this case, electron and lattice temperatures would differ). Contrary to these expectations, some of the conditioned metal thin films showed the emissivity quite comparable with the emissivity of carbon films in [38,39,40]. Hence, the emission model should be corrected to explain the observed indistinction.

### 4.2. On the Emissiom Mechanism

We propose a revised emission mechanism illustrated by the diagram in Figure 10b. Its basic elements were previously considered in our article [62] and in earlier works by Fedorovich et al. [29] on the surface-current-induced emission—in both cases, among the less plausible options. As in the previous case (Figure 10a), electric field of a charged islet (in combination with a weaker field of the anode) caused tunneling of electrons from the rim of the main film body. However, in the revised model (Figure 10b), electrons are transferred into the vacuum region adjacent to the islet and proceed to the anode. The passing electrons produce electric polarization of the islet. The time-dependent component of this polarization may be described in terms of localized plasmonic oscillations. For the involved sort of nanoislets (refractory metals or carbon), plasmon decay most probably results in the release of heat, which is necessary in the considered thermoelectric model to maintain the islet’s positive potential. (The very possibility of plasmonic oscillations, induced by emission of single electrons by metal islets, has been previously considered, e.g., in publications [27,107]). Electroluminescence from the emission area registered in some experiments can be attributed to electromagnetic decay of the plasmons [106].

Plausibility of the model can be assessed by the following order-of-value numeric estimates. Energy transfer from tunneling electrons to plasmons in single-electron processes are often considered as a consequence of statistical or “shot” noise component in the emission current, as it has been done, for instance, in [108] (for the basic theory, see references therein). Shot noise is known to have uniform (“white”) spectral distribution up to the frequencies determined by the shape of single-electron pulses induced in the “load.” In our case, they can be estimated from spatial dimensions of the problem defined by the size of the smallest islets seen in microscopic images, 1–10 nm. In the classical approach, electrons with energies 1–10 eV (the expected values of the islet potential) have velocities of the order of 10^6^ m/s; thus, the typical flight times are 10^−15^–10^−14^ s. The corresponding frequencies lie in the waveband of localized surface plasmon resonance [109], which must result in efficient electron-plasmon interaction. For a single electron, the energy of interaction with an islet can be roughly estimated from the formula for Coulomb interaction of a charge with a conductive plane:

(1) $$\Delta E=\frac{e^{2}}{4{\pi \varepsilon }_{0}\cdot 2b},$$

where b is the minimal distance between the electron trajectory and the plane (the islet). For 2b = 10 nm, the formula gives ΔE ≈ 0.15 eV. A typical value of current from a single emission site 10 μA corresponds to the emission rate of the order of 10^14^ electrons per second. Multiplying this value by ΔE, we obtained an estimate of 2 μW for the power that may be transferred by emitted electrons to plasmonic oscillations in an EC islet (“B” in Figure 10b) to be further released as a heat. Even for a relatively large islet/substrate interface area, 10 × 10 nm^2^, the resulting heat flux density is as high as 2 MW/cm^2^. According to the previous estimates [62], this may be sufficient for thermoelectric maintenance of the islet positive potential as high as several Volts.

It might be noted that the performance of the emission mechanism in its revised version (unlike the original one) practically does not depend on details of electronic structure of the islets—it requires only the ability to support plasmonic oscillations converting their energy into the heat (i.e., to be larger than 1–2 nm in size [110]). The assumption of size-effect-reduced hot electron relaxation rates (contested in the mentioned works [100,101]) is not necessary in this version of the model. The thermoelectric potential is generated mainly outside the islet—in a crystalline substrate in its vicinity. Therefore, the emission capability of a film may be less sensitive to its material than to the islands’ size and to the interfaces. The observed film material dependence of the emission properties can be explained by different patterns of film agglomeration in the conditions of TF treatment and emitter operation [63]. Apparently, the employed TF forming procedure proved to be optimal for the conversion of the solid Mo films into the most effectively emitting structure. The structure consists of regions where the film had disintegrated into separate nanoparticles, alternating with areas, where the film retained its continuity, which secured high electric conductance.

### 4.3. Nanodot Emission Cell as Object for Further Studies

According to the presently dominant opinion on the phenomenon of low-field electron emission from the smooth emitting boundary, it cannot be exploited in technological applications. In diode schemes, such smooth-surface emitters cannot provide high emission current, their behavior is unpredictable, and the current is instable and originates from a very limited number of ECs. The ECs morphology and concrete emission mechanism have avoided reliable definition for many decades, but some experiments with carbon species demonstrated that the emission mechanism might still be associated with high field enhancement [111] and that hardly detectable high-β carbon fibers [90,91] might be present at such surfaces and can be responsible for the LMF emission. The results of our experiments reported in this and previous papers [38,39,40] partially confirm this position—at least regarding the difficulty of achieving a large total emission current, its instability, and low density of emission centers. However, they also witness in favor of an emission mechanism different from the trivial β-enhanced FN emission. In particular, the observed similarity in emission properties and in the optimal activating treatment procedures for thin films of carbon and molybdenum contradicts the idea of associating the LMF emission phenomenon with the formation of high-aspect protrusions—because Mo whiskers are less readily formed and are much easier detected in SEM images (and were never detected in the hundreds of images made for emitting regions). Therefore, we associated the acting ECs with the nanoislets that were observed in all our studies, for both carbon [38,39,40] and metal films, and we suggest the alternative emission model.

**Figure 11:**
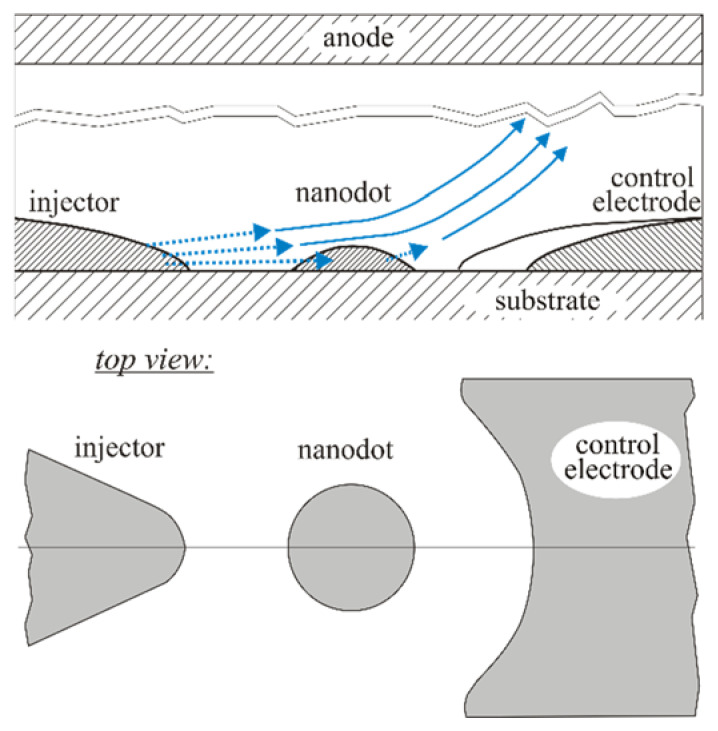
Scheme of the suggested emission unit.

Yet, for practical applications, it is important that the ECs of the suggested type could have appeared only due to a random combination of factors. This determines a large scatter of their characteristics and relatively low area density, which prevented achievement of more satisfactory operational parameters. A way to significantly improve operational parameters of such emitters may consist of the fabrication of ordered arrays of nanodot-based emission units schematically shown in Figure 11—one of the suitable manufacture technologies is described in [112,113]. Geometrically, this unit is similar to the one proposed by Fedorovich et al. [30], yet it uses a different operational principle. The main element of the proposed scheme is a nanoislet (or a nanodot) employed for the extraction of the emission current from the injector electrode through the action of a self-sustained electric charging, in accordance with the suggested mechanism. The control electrode serves to direct the emitted electrons to the anode. It can also be used for emission current initiation and/or quenching.

The presented design may be regarded as an analogue of the Spindt-type emitter scheme [10,11], with the possible advantage of a better durability. In the Spindt’s system, the emitting spot is located on the tip, which implies hindered heat dissipation. In the proposed design, the emitting area has good thermal contact with the substrate—the corresponding thermal problem has been considered in papers [30,33]. Furthermore, the emission current in the proposed scheme is explicitly determined by the thermoelectric potential and thus by the islet lattice temperature. Therefore, the temperature can be controlled via a current control circuit. Manufacture and testing of such a cold emission unit may represent a promising objective for further studies.

## 5. Conclusions

In the reported experiments, we studied field emission properties of thin (6–10 nm) films of molybdenum deposited by magnetron sputtering onto naturally oxidized flat silicon substrates. After forming by heating in an electric field, the samples produced a measurable room-temperature emission current at very low macroscopic field magnitudes—starting from 1.4–3.7 V/μm. In the literature, room-temperature emission from metal thin films deposited on dielectric layers was often reported to occur under the action of a driving electric current. In our experiment, it was caused solely by the action of an electric field applied to a vacuum boundary, as in a conventional vacuum diode.

Microscopic studies of the samples performed before and after emission experiments have shown the absence of any visible high-aspect surface features that could substantially facilitate electron emission by geometric field enhancement. However, the forming procedures and emission testing induced partial dewetting of the initially continuous films, which led to the appearance of numerous areas where the Mo layer comprised separate nanoislets. We associated the observed electron emission with such areas, as the LMF emissivity is known to be an inherent property of many electrically nanostructured heterogeneous materials and films.

Comparison of the present results with previous data for carbon islet films of nm-scale effective thickness formed on identical substrates showed their similarity in the basic emission parameters, such as threshold field values and FN emission characteristics’ slopes. This fact witnesses in favor of a common emission mechanism for discontinuous films of carbon and metals. We are proposing a novel model of LMF electron emission from such islet films (either carbon or metallic), representing a combination of the patch-field, multiple-barrier and thermoelectric emission models suggested in the literature for different ENH materials.

## Data Availability

The data presented in this study are available on request from the corresponding author.
